# Adult Wilms' tumour: a case report with review of literature

**DOI:** 10.1186/1746-1596-1-46

**Published:** 2006-12-05

**Authors:** V Geethamani, V Kusuma, KM Srinivasa Gowda, Monika Lamba Saini

**Affiliations:** 1 Department of Pathology, Kempegowda Institute of Medical Sciences, Bangalore – 560004, India; 2 Director, Kempegowda Institute of Medical Sciences, Bangalore – 560004, India

## Abstract

**Background:**

Wilms' tumor is the commonest primary malignant renal tumor in childhood. Rarely, it may present in the adult age group.

**Case presentation:**

We report a 48-year-old male presenting with flank pain and haematuria. Abdominal ultrasound revealed a right renal mass measuring 11 × 10 cms, and a clinical diagnosis of renal cell carcinoma was made. Nephrectomy was performed, and a final diagnosis of adult Wilms' tumor was made based on the criteria proposed by Kilton *et al*.

**Conclusion:**

The possibility of an adult Wilms' tumor should be considered when a patient presents with pain in the flank and a renal mass. Rarity of the tumor favors documentation in literature.

## Background

Nephroblastoma or Wilms' tumor is the most common malignant renal tumor in children. It accounts for approximately 5–6% of the neoplasms in children and is rare in the adult population [1]. Less than 3% of all the reported Wilms' tumor cases occur in adults. The overall survival to the tune of 83% has been recently reported with the use of primary nephrectomy followed by adjuvant combination chemotherapy [2].

## Case report

A 48-year-old male presented with haematuria and dull aching pain in the right flank. The general examination of the patient was unrevealing, with no lymphadenopathy or bony tenderness. The chest, cardiovascular and neurological examination was normal. Abdominal examination revealed a mildly tender mass palpable in the right lumbar region. Routine laboratory investigations including a full blood count, chest X-ray, and renal function tests were normal. Urine examination showed plenty of RBCs in high power field.

An ultrasound examination of the abdomen revealed an echogenic mass in the upper part of the right kidney measuring 11 × 10 cm. No calcified areas were noted; the renal pelvis and renal vein were not involved. The other abdominal viscera were radiologically normal. There was no lymph node enlargement. A provisional diagnosis of renal cell carcinoma was made and right radical nephrectomy performed.

Grossly, the nephrectomy specimen weighed 480 grams and showed a solid spherical tumor measuring 10.5 × 10 cm. The cut surface was variegated with grey white, fish flesh appearance. The adjacent renal parenchyma appeared normal. The renal capsule was intact. There was no involvement of the renal pelvis or vein.

**Figure 1:**
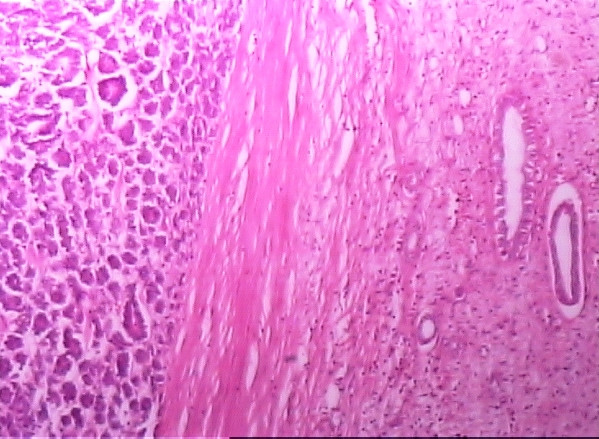
Triphasic pattern showing tubules, solid sheets of cells, and stromal differentiation.

**Figure 2:**
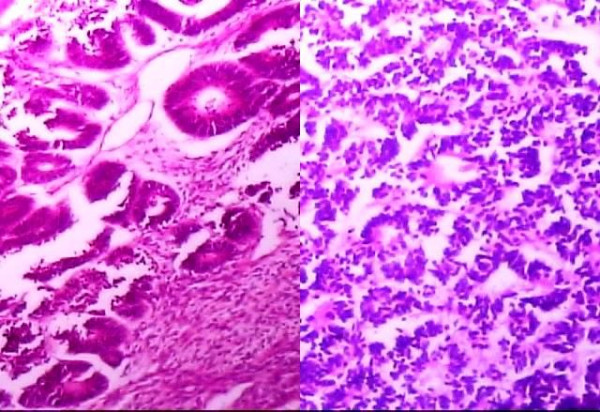
Tubular pattern resembling pseudo-rosettes at places.

Microscopically, the tumor was highly cellular comprising of epithelial, blastemic and stromal elements. The epithelial element comprised of tubules admixed with blastemic cells (Fig. 1). The tubular lining cells had elongated, molded, and wedge shaped nuclei exhibiting frequent mitoses. The tubular pattern resembled pseudo-rosettes at places (Fig. 2). The blastemic component had solid areas showing cells with oval nuclei and scanty cytoplasm. The stroma with spindle cells had no specific differentiation. There was no area showing anaplasia. A final diagnosis of adult Wilms' tumor with triphasic pattern and a favorable histology, stage 2, was made.

Adjuvant treatment was started as per Intermediate Risk protocol for Stage I Node negative disease with vincristine 1.4 mg/m^2 ^and actinomycin D 15 mcg/kg. The patient remains in complete remission after 14 months of follow up.

## Discussion

Wilms' tumor, named after the 19^th ^century German surgeon Carl Max Wilhelm Wilms, is probably derived from primitive metanephric blastema. The histological appearance is characterized by marked structural diversity. Classic Wilms' tumor is composed of three types of cells – blastemal, stromal, and epithelial; although the occurrence of all three types in the same case is uncommon [3].

Adult Wilms' tumor is diagnosed based on the criteria given by Kilton, Mathews, and Cohen [4]. These include 1) the tumor under consideration should be a primary renal neoplasm; 2) presence of primitive blastemic spindle or round cell component; 3) formation of abortive or embryonal tubules or glomerular structures; 4) no area of tumor diagnostic of renal cell carcinoma; 5) pictorial confirmation of histology and 6) patient's age >15 years. Kilton et al (1980) reported 35 cases of adult Wilms' tumor complying with all the above criteria.

To the best of our knowledge, the present case is only the second case in Indian literature to have the classic triphasic histology. Blastemic component was predominant in the histology in 6 out of the 8 adult Wilms' reported in Indian literature [5,6].

The differential diagnosis of an adult Wilm's tumor with mainly epithelial differentiation includes metanephric adenoma. A predominant blastemic Wilms' tumor has a strong resemblance to small, blue round cell tumors which commonly include lymphoma, peripheral neuro-ectodermal tumor and rhabdomyosarcoma; and rarely metastatic small cell tumors from lung, immature teratoma, and primary renal cell sarcoma. Extensive search for any other components is needed as a poorly differentiated renal carcinoma can have large sarcomatous areas resembling blastema [4].

Most adults present with local pain and haematuria, in contrast to the palpable boggy mass which is more common in children. In adults, Wilms' tumor is larger and ill-defined, with areas of necrosis and hemorrhage. About half of the patients have stage 3 or 4 disease [7]. There are about ten cases of extra-renal Wilms' tumors in adults documented in the literature. Four of them were in the retroperitoneal region, two each in the ovary and endometrium, and one each in ovotestis and prostate [4].

Tremendous data on the biology and epidemiology of Wilms' tumor has been generated by the National Wilms' Tumor Study (NWTS) which was established in the USA in 1969. An update from the NWTS group about treatment outcomes in adults with favorable histology Wilms' tumor (FHWT) described 45 patients treated in the modern era. The overall survival rate was 82% [8].

In 2004, Reinhard et al reported their experience with 30 cases of adult Wilms Tumor [2]. A complete remission was achieved in 24 of their patients. Event-free survival was 57%, and overall survival was 83%. They concluded that adults can be cured in a high percentage by a multimodal treatment according to pediatric protocols.

Wilms' tumor in adults has a worse prognosis than in the pediatric population, a phenomenon for which there is no adequate explanation [1]. As adult Wilms' tumor is rare, randomized trials cannot be performed. It has been suggested by most authors that to evaluate concepts for adequate treatment, results of randomized trials with childhood Wilms' tumor should be extrapolated. National Wilms' Tumor Study (NWTS) and other studies have recommended multimodal therapy for the disease with surgery, chemotherapy (actinomycin D, vincristine and doxorubicin) for 15 months and tumor bed irradiation in the case of stage 3 disease. Less aggressive therapy with two drugs is advised in stage 1 and 2 disease.

## Conclusion

The possibility of an adult Wilms' tumor should be considered when a patient presents with pain in the flank and a renal mass. Although the prognosis is poorer than that of children when the disease is compared stage for stage, the outcome for adult patients diagnosed with Wilms' tumor is steadily improving.
